# Linking Older Adults’ Psychosocial Well-Being With Objective and Perceived Environments in Slovenia

**DOI:** 10.1177/19375867251343909

**Published:** 2025-06-25

**Authors:** Mateja Erce Paoli, Michael D. Burnard

**Affiliations:** 1 682164 InnoRenew CoE, Izola, Slovenia; 2 Andrej Marušič Institute, 244385 University of Primorska, Koper, Slovenia; 3 Faculty of Agriculture and Forestry, University of Helsinki, Helsinki, Finland

**Keywords:** needs, retirement homes, successful aging, environmental quality, structural equation modeling

## Abstract

**Background:** Europe's aging population faces resource loss, loneliness, and social isolation, making psychosocial well-being a priority. A well-designed environment can help compensate for lost resources, while poor design may harm health, highlighting environmental quality as key to successful aging. **Aim:** The aim of our study was to evaluate the link between psychosocial well-being, and the built environment. **Method:** Participants from three retirement homes in Slovenia filled in the World Health Organization Wellbeing Index, the Brief Sense of Community, the Lubben Social Network Scale, the University of California Los Angeles Loneliness Scale, and the short version of the Social Production Function Instrument with the assistance of a researcher. Additionally, the built environment was objectively assessed using the EVOLVE tool and subjectively assessed by participants using a custom scale. Structural equation modeling was used to examine paths between the environment and wellbeing. **Results:** The findings showed a statistically significant indirect effect of well-being on the perceived environment, while no indirect effects were found between the objective and perceived environment or well-being. However, specific objectively measured environmental factors, such as acceptability, features for sensory and dementia support, and perceived accessibility and aesthetic environmental attributes significantly affect well-being. **Conclusion:** These findings highlight the need to prioritize well-being in urban planning for aging populations. Beyond basic needs like safety, high-level needs like aesthetics, sensory support, and accessibility should be emphasized. Enhancing well-being through these factors may be effective when designing or modifying physical environments. Accessible, sensory-friendly, and dementia-supportive designs can further support healthy aging.

## Introduction

The built environment is a critical aspect of health and well-being—particularly for aging populations living in urban areas, where people spend around 85% of their time indoors (Sattari et al., 2023). Understanding the environment's contribution to satisfying occupants’ psychosocial needs is an important mode of investigating the link between well-being and the built environment. Maintaining functional abilities, which a well-designed built environment can support, contributes to well-being and enables healthy ageing. The role of the built environment is especially significant in the case of retirement homes, which serve as important locations in many older adult's lives. Transitioning to a retirement home comes with relocation, potential loss of meaningful relationships, new daily routines, an introduction to a new community, and change of environment, all of which impact many dimensions of well-being and should be assessed together (Elo et al., 2011; Tang et al., 2017).

Psychosocial well-being—a combination of psychological or emotional well-being, social, and collective well-being—is an appropriate and important factor for identifying beliefs, expectations, sense of community and belonging, which are all influenced by built environments (Eiroa-orosa, 2020; Elo et al., 2011). As well-being and sense of community are linked to needs’ satisfaction, improving the built environment is crucial (Pincus, 2024; Ryff, 2014; Tang et al., 2017, 2018). Assessing both subjective experiences and objective measures of the environment helps to mitigate biases in evaluating therapeutic or health environments (Gesler et al., 2004; Pontin et al., 2022). Additionally, the perceived environment can mediate the link between well-being and observed environmental quality (Evans et al., 2002).

### The Built Environment Affects Older Adults’ Quality of Life

Environmental features have been linked to well-being and mental health. Features that encourage physical activity can also improve mental health, whereas a lack of features that support mobility, like ramps, can lead to social disconnection (Bhuyan & Yuen, 2021; Cao et al., 2020; Guo et al., 2021; Van Dyck, 2015). Broader quality of life has been linked with balances between private and public space, and how homelike an environment is (Fleming et al., 2016; Hua et al., 2022). Research shows that older adults’ needs can be addressed by features of the environment, e.g., safe paths in parks support social needs, and vice versa—parks as social venues affect physical activity as a basic need (Garvin et al., 2012). While research has focused on outdoor environments, indoor environments play a crucial role in older adults’ lives based on amount of time spent indoors (Azim et al., 2023). Air quality indicators like temperature and level of pollutants have been identified as most critical in studies that focused on indoor environments, while the visual environment and illuminance were studied less, highlighting the need for broader assessment of interior environments (Fleming et al., 2016; Reddy et al., 2021; Zhan et al., 2021).

Key environmental factors promoting social well-being include age-friendly environments, adaptable spaces, green and blue areas, homelike atmospheres, and features supporting independence and safety (Finlay et al., 2021; Gibney et al., 2020; Hongyun, 2013; Marquardt et al., 2014; McIntyre & Harrison, 2017; Miller & Kälviäinen, 2012; van Hoof et al., 2017). While age-friendly cities are crucial for aging in place, the effectiveness of environmental interventions remains uncertain due to methodological challenges (Sánchez-González et al., 2020; Sterns et al., 2020). Research highlights cultural differences in age-friendliness—high residential density fosters community in Asia, spatial legibility and street connectivity enhance well-being in Europe, and social infrastructure supports cognitive health in the U.S. (Finlay et al., 2021; Guo et al., 2021; McIntyre & Harrison, 2017; Nygaard et al., 2022; Sterns et al., 2020). To ensure the relevance of environmental interventions that support ageing, it is essential to involve older adults in urban planning (Black & Jester, 2020). For a more detailed discussion on interventions, see Van Hoof & Marston (2021).

### The Conceptual Model

**Figure 1.**
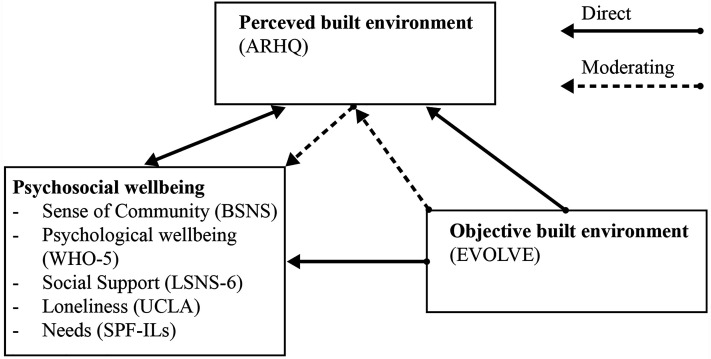
Conceptual model applied in this study. *Note.* Total sums of questionnaires were used in the statistical analysis. For definitions of terms, see Table S4 in Supplementary materials.

Our conceptual model (Figure 1) is based on previously established research and reviews (Brown et al., 2009; Buckley, 2022; Evans et al., 2002; Firdaus, 2017; Orstad et al., 2017; Padeiro et al., 2021; Van Dyck, 2015; Wang et al., 2019; Yue et al., 2022; Zhang et al., 2018) including Guo et al.’s (2021) model, which examined the relationship between objective and perceived built environments in relation to sense of community and mental well-being. However, their study was limited to the external environment. Our model relates the objectively measured indoor built environment's influence on the well-being of older adults through their perception of an environment, with a reciprocal relationship between subjective evaluations of the environment and well-being.

### Objectives

The study aimed to identify connections between the physical environment and psychosocial well-being, while also analysing the relationship between perceived and objective measures of the built environment.

## Materials and Methods

### Study Location and Participants

This study was conducted in the coastal region of Slovenia in three retirement homes (to maintain anonymity, referred to as: A, B, C, for more information, see Table 1). The eligible area was within 5 km of the coast, in an urban area, and with signed informed consent from directors. Participants residing in these retirement homes were recruited in person. Survey data were collected through face-to-face interviews between December 2023 and April 2024. Among 195 eligible residents aged 60 and over, 134 signed an informed consent to participate and were interviewed.

**Table 1.** Characteristics of Selected Retirement Homes.

Retirement home	Location	Nu. of residents	Year of construction	Distance from the coast	Nu. of retirement homes in the area	City population
A	Historical part	205	1953	0.3 km	1	16,430
B	Residential area	150	2013	2.6 km	2	26,305
C	Coastal urban area	164	2008	0.5 km	1	6,974

*Note.* In the area where Retirement Home B is located, only one retirement home was included in the study, as the director of the other home did not sign an informed consent.

Participants were eligible to participate if they were (1) older adults, living permanently in a retirement home, (2) had the ability to walk independently, with or without a walking aid, (3) and the ability to converse in Slovenian. Exclusion criteria included severe psychiatric disorder or progressive neurological disorder (except mild dementia), and severe visual impairment.

### Ethics

Ethical approval was obtained from The National Medical Ethics Committee of the Slovenia board prior to conducting the study [0120-343/2022/5]. Informed consent was obtained from all participants, emphasizing voluntary participation, confidentiality, and the right to withdraw from the study at any time.

### Measures

The Clock Drawing Test was used as an initial screening for potential cognitive impairment. This is a short instrument where a participant needs to draw a circle on a piece of paper and then draw the hands at a given time. The instrument has shown satisfactory psychometric properties and has been tested in a population of Slovenian older adults (Avberšek et al., 2005; Shulman, 2000).

#### Psychosocial Well-Being

To measure psychosocial well-being holistically, we included five questionnaires that were validated on a population of older adults: The five-item World Health Organization Wellbeing Index (WHO-5; Eser et al., 2010; Lucas-Carrasco, 2012) for evaluation of psychological well-being; the eight-item Brief Sense of Community Scale (BSCS; Buckley, 2022; Sočan & Kobal Grum, 2023; Yu et al., 2019) for individual sense of community; the nine-item Lubben Social Network Scale (LSNS-6; Daniel et al., 2023; Lubben et al., 2006) and the six-item University of California Los Angeles Loneliness scale (ULS-6; Neto, 2014) for evaluation of social well-being; and the 15-item Social Production Function Instrument (SPF-ILs; Nieboer & Cramm, 2018) for evaluation of needs.

We adapted the LSNS-6, adding duplicates of three questions replacing the words “relatives” and “friends” with “employees in the retirement home” to acknowledge the important role retirement home employees may have in a resident's social network. These additions were not formally validated.

#### Perceptions of Retirement Homes

As no instruments were available to gather subjective evaluations of retirement home interiors, we developed a new instrument for that purpose. The Aging in a Retirement Home Questionnaire (ARHQ) was designed from a review of existing tools and interactions between built environments and older adults needs (Erce et al., 2022; Erce Paoli & Burnard, 2024). The first author then translated the questionnaire to Slovenian while the second author checked the translation. At the final stage, authors harmonized the translation. This instrument has not yet been validated.

The ARHQ consists of 37 items, divided to two parts: (1) 32 items focusing on the respondent's room, and (2) five items to evaluate retirement home in general. A five-point Likert scale was used, with response options ranging from 1 (*completely disagree*) to 5 (*completely agree*), where higher values indicated greater agreement. The sum of all items was used in path modeling. A factor structure was also tested for this instrument, with the best fit structure at four factors (minimum MAP and empirical BIC reached at four factors with other selection methods unclear; oblique rotation and ordinary least squares factoring). The factor structure fit was moderate (KMO = 0.77), 11 items were not included in any factor (Tables S1 and S5, Supplementary materials).

#### Objective Built Environment

The EVOLVE tool—evaluation of older people's living environments—was used to assess each respondent's room and each retirement home (Lewis et al., 2010). Of EVOLVE's many components we used only the (double or single) bedroom (64 or 61 items), bathroom (81 items), and hall (32 items). This resulted in a total of 174–177 items, depending on whether the person was living with roommates or had their own room (Table S2, Supplemental materials).

#### Covariates

Based on previous studies, we included several covariates in our model (cf. Dong et al., 2014; Golden et al., 2009; Lee & Tan, 2019; Litwin & Stoeckel, 2013; Tang et al., 2017). These were: age (categorical, bracketed into seven levels), reason for choosing to move to a retirement home (categorical, seven levels), time to adapt to the retirement home (ordinal, six levels), number of chronic diseases (count), months lived in the retirement home (continuous), gender (binary), educational level (categorical, nine levels), marital status (categorical, six levels), number of roommates (count), type of building lived in most of their life (categorical, four levels), residency location (categorical, three levels), and income (categorical, five levels). On the building level, we recorded sky orientation (categorical, four levels), having a balcony (binary), having a private bathroom (binary), and number of plants in a room (count) (Table S3, Supplementary materials).

Self-reported health and one's perception of one's own health were measured using the ED-5Q-3L and EQ VAS (Buchholz et al., 2022).

### Procedure

Participants were given a printed scale on a separate sheet for each questionnaire. The researcher read each item aloud, and participants responded either verbally or by pointing to their answer on the sheet following the method used by Triadó et al. (2007).

A potential bias in this study is the uneven timing of data collection across the three retirement homes. Data was collected first in A between December 2023 and February 2024, and then in B and C between February and April 2024. This uneven timing could introduce bias related to external factors, such as changes in health and well-being status, seasonal effects, or other temporal influences.

### Statistical Analysis

Data were analyzed in RStudio 2024.04.2 (Posit Team, 2024), using R version 4.3.2 (R Core Team, 2024). In R, we used the lavaan package (Rosseel, 2012), psych package (Revelle, 2024), mice package (Van Buuren & Groothuis-Oudshoorn, 2011), and tidyverse family of packages (Wickham et al., 2019). First, we calculated the sample's descriptive statistics. Second, we conducted an exploratory factor analysis (EFA) and confirmatory factor analysis (CFA) of the ARHQ. Multilevel structural equation modeling (SEM) was used to examine paths from objective and perceived built environment to well-being as well as for exploring effects of the environment on well-being. Numerical values were scaled to be between 0 and 1 to ensure variances were comparable, unordered categorical data were coded as dummy variables, while ordered categorical data were coded numerically, and treated as continuous values. The unit of analysis was the individual respondent (*n* = 119 complete observations).

We compared two SEM models that examined paths from environment to well-being, the first with a fuller set of co-variates (Model 1) and the second with a reduced set (Model 2). In both cases, well-being was treated as a latent variable consisting of psychological well-being (WHO-5), individual sense of community (BSCS), social well-being (LSNS-6, with our additions), and needs (SPF-ILs). To construct a reduced model, we proceeded with a stepwise removal of variables that had little influence on the model (*p* > .05). The final reduced model contained having a balcony, living environment, reason for choosing to move to a retirement home, number of diseases, adaptation time, type of environment, and marital status as covariates. We chose the best fitting model, Model 2, which showed a good fit (χ^2^ (65) = 100.266, CFI = 0.889, TLI = 0.829, RMSEA = 0.068, SRMR = 0.037).

As dummy variables were used in the models, the following levels of these variables were considered baseline in the final model (i.e., not given a dummy coding, or when all other levels are set to 0): reason for moving to the retirement home: *health status*, living environment for most of one's life: *city*, type of building one lived in for most of their life: *single-family home*, marital status: *married*, retirement home: *A.*

To determine the attributes of the indoor environment that can be linked to well-being, a SEM was conducted using the selected EVOLVE domains as predictors of well-being (Model 3). Model 3 showed a good fit (χ² (38) = 48.993, *p* = .000, CFI = 0.930, TLI = 0.900, RMSEA = 0.049, SRMR = 0.036). Additionally, a separate SEM was conducted using ARHQ subscales as predictors of well-being. The first model (Model 4) focused on respondent's rooms and showed a moderate fit (χ² (276) = 389.365, *p* = .000, CFI = 0.854, TLI = 0.831, RMSEA = 0.073, SRMR = 0.074). The second model (Model 5), which analyzed the overall retirement home environment, indicated a marginal to poor fit (χ² (18) = 40.806, *p* = .002, CFI = 0.911, TLI = 0.861, RMSEA = 0.103, SRMR = 0.061).

## Results

### Sample Characteristics

The population of eligible adults, as defined by social workers in each retirement home, was 195 persons, with a sample of 134 older adults, from which 119 participants provided complete responses. Of these, 64.7% were women (0% nonbinary), 68.9% were widowed, and 73.9% had at least secondary education. Residents had lived in retirement homes for a median of 15 months (IQR: 30 months), half lived with one roommate (50.4%), 42% lived alone, and 7.6% had two or more roommates. The main reasons for entering were health issues (59.7%), desire for an orderly life (25.2%), and loneliness (5%). Adaptation times varied: 46.2% adapted in under a month, 17.6% in 1–6 months, 11% in over 6 months, and a quarter had not yet adapted. Notably, 10% of those who had not adapted had been in a retirement home for 6 months or less. Nearly 30% had no chronic illnesses, 38.7% had one, 20.2% had two, and the rest had three or more. Most had lived in cities (70.6%) for the most of their life, with 46.2% in single-family homes, 42% in apartments, and the rest in multi-apartment houses. Financially, 31.9% felt their income matched their peers, 25.2% felt it was slightly lower, 22.7% felt it was slightly higher, and the rest felt it was much lower or higher.

Adaptation times to retirement homes were analyzed across user profiles. While they do not clearly indicate distinct groups, the data shows that most individuals adapted relatively quickly (within 1 month), while a notable portion has not yet adapted. The distribution of adaptation times between these extremes is relatively flat and low. For example, 43% of women adapted within 1 month, while 27% had not yet adapted. Among men, 52% adapted within 1 month, whereas 21% had not yet adapted. In both cases, between 27% and 30% adapted at some point between these extremes.

### Well-Being as a Latent Variable

Well-being was treated as a latent variable constructed of psychological well-being, needs satisfaction, sense of community, social network and loneliness. The factor loading of the latter indicated a weak contribution to well-being and was excluded. The other items were indicated as suitable measures of the psychosocial well-being construct (Table 2).

**Table 2.** Results of EFA.

Indicator	Factor loading	Communalities	Unique variance
WHO-5	0.83	0.697	0.303
Soc. Network	0.47	0.221	0.778
Sense of community	0.65	0.421	0.579
Needs	0.76	0.580	0.420

*Note*. χ^2^ (6) = 118.676, TLI = 0.969, CFI =0.990, RMSEA = 0.076, SRMR = 0.031.

### Structural Equation Modeling Results

To examine the relationships between the built environment and well-being, several SEMs were conducted, each focusing on different aspects of these interactions.

#### Predictors of Well-Being Based on Our Conceptual Model

**Figure 2.**
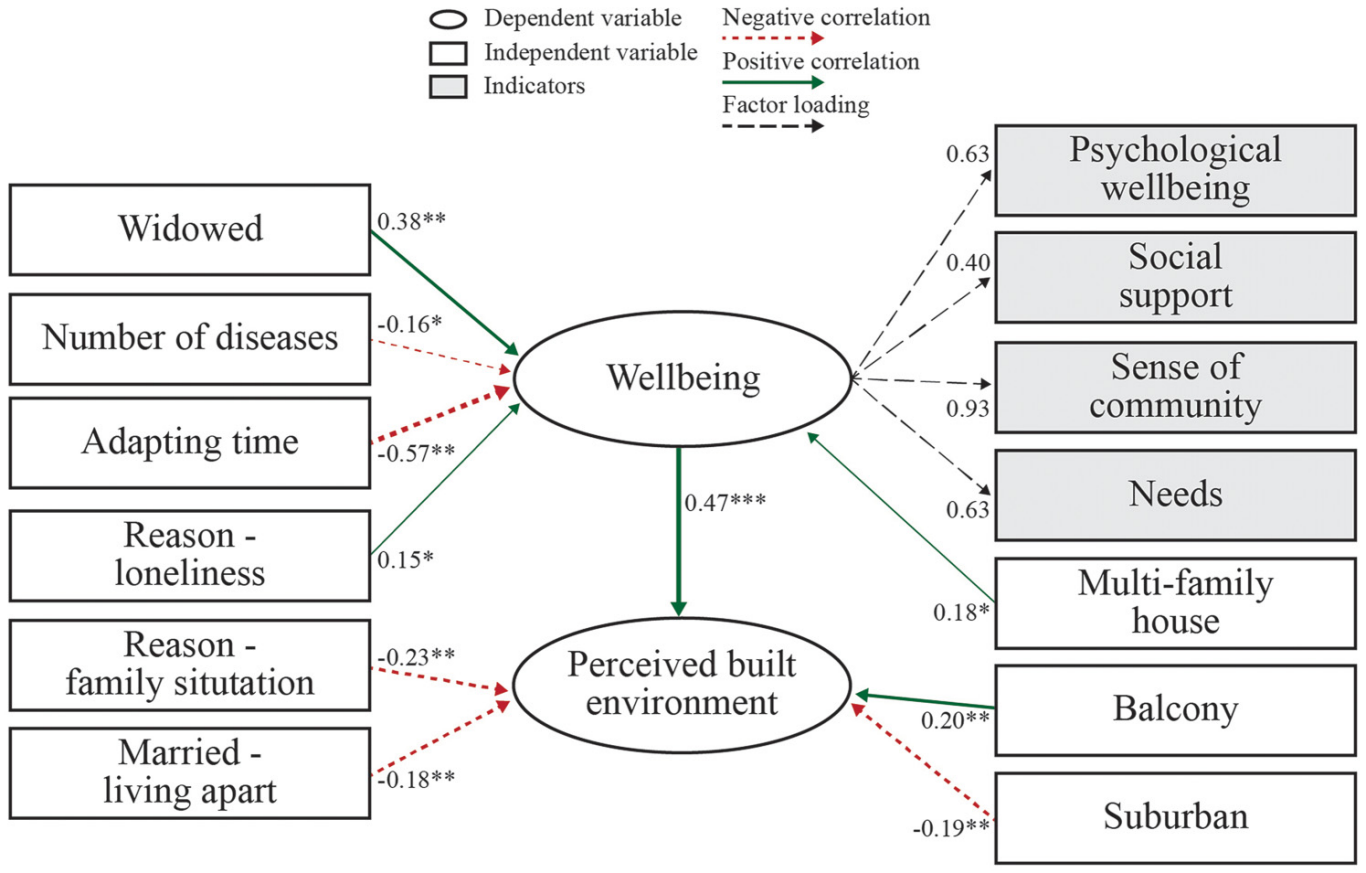
Path diagram for the hypothesized model derived by SEM. *Note*. *** *p* < .001; ** *p* < .01; * *p* < .05. See Table S4 in the supplementary materials for definition of terms. A full definition of all terms is available at [Zenodo: https://doi.org/10.5281/zenodo.13865692].

Model 2 showed that a higher number of diseases (β = .010, *p* = .022) and longer adapting time (β = .007, *p* < .000) had a negative effect on well-being, while living in a multifamily housing most of one's life (β = .037, *p* = .017), going to a retirement home due to loneliness (β = .055, *p* = .044) and being widowed (β = .047, *p* = .006) had a positive effect on well-being, with objective and perceived environment having insignificant effects. Having a balcony (β = .029, *p* = .006) had significant positive effects on one's perception of the built environment. Of the reasons for moving to a retirement home, only unsettled family situations (i.e., domestic violence, social distress, disputes) was significant (β = .057, *p* = .008) and had a negative effect on perceived built environment. Having lived in a suburban environment most of one's life also had a negative effect on their perception of their retirement home (β = .51, *p* = .005). Similarly, among all marital statuses, only being married but living apart had a significant effect (β = .149, *p* = .005) (Figure 2).

The indirect effect of the objective built environment on well-being through perceived built environment was not significant (β = .023, *p* = .785), and there was no statistically significant relationship (β = .085, *p* = .099) where both the objective built environment and well-being influence perceived environment. Similarly, the objective built environment did not directly affect the perceived environment (β = .124, *p* = .069), while well-being affected subjective evaluation of an environment (β = .149, *p* = .000). However, objective and perceived built environment did not influence well-being (β = .046, *p* = .677 and β = .101, *p* = .783, respectively). Additionally, there was no significant total effect (β = .096, *p* = .184) (Table 3).

**Table 3.** Estimates of Direct, Indirect, and Total Effects Between the Built Environment and Well-Being.

	Direct effects		
	Coef.	Std. Error	Z-value
OBE → PBE	0.225	0.124	1.821
OBE → WB	−0.019	0.046	−0.416
PBE → WB	0.028	0.101	0.275
WB → PBE	0.623	0.149	4.191***
	Indirect effects		
OBE → PBE →WB	0.006	0.023	0.273
OBE → PBE ← WB	0.140	0.085	1.649
Total effect	0.128	0.096	1.327

*Note*. OBE = objective built environment, PBE = perceived built environment, WB = well-being; *** *p* < .001; ** *p* < .01; * *p* < .05; the arrow sign (→) indicates the prediction of variables.

#### Impact of the Indoor Environment on Well-Being

The SEM identifying the impact of the subscales of objective environment on well-being (Model 3) showed that acceptability (β = −.530, *p* = .043), sensory support for sight, hearing, touch, smell (β = .574, *p* = .013), and dementia support (β = −.636, *p* = .010) had significant effects on well-being. Another SEMs, identifying the effects of the subscales of the perceived built environment on well-being (Models 4 and 5) showed that a esthetic qualities of a room significantly affected well-being (β = .874, *p* = .000), as well as accessibility of a retirement home (β = .348, *p* = .004), while room's visual qualities, indoor air quality, and control over the environment did not.

## Discussion

The aim of our study was to test a conceptual model designed through an extensive review of the literature, focusing on the relationship between the indoor built environment of retirement homes and the psychosocial well-being of resident older adults. We hypothesized that the subjective evaluation of retirement homes by older adults would mediate the relationship between the objectively measured environment and their psychosocial well-being. Similarly, we predicted that there will be a positive correlation between objectively and subjectively measured built environment, as well as between objective and perceived environment and well-being.

### Predictors of Well-Being Based on Our Conceptual Model

When considering the total sum of variables, our model results did not align with our predictions. There were no significant relationships between perceived and objective environment, nor was the objective environment found to influence well-being through subjective evaluation of the environment. Similarly, the perceived environment did not impact well-being. However, we found the reverse relationship to be significant, well-being significantly influenced the respondent's perception of the environment.

*However, we found the reverse relationship to be significant, well-being significantly influenced the respondent's perception of the environment.*

Our findings are in contrast with Guo et al. (2021)'s study, who found that subscales of the objective environment were related to both the perceived environment and mental well-being. Guo also demonstrated that the objective environment influenced mental well-being through the perceived environment as a mediator. Similarly, Zhang et al. (2018) reported that objective accessibility to daily living services was associated with mental well-being through the perceived environment. Furthermore, objectively evaluated housing quality has been shown to influence psychological well-being through place attachment, and the relationship between the objective environment and psychological distress has been mediated by perceived social support (Brown et al., 2009; Evans et al., 2002).

In contrast to our results, other studies have also highlighted significant links between objective environmental factors and mental well-being (Evans et al., 2002; Firdaus, 2017; Yue et al., 2022).

Our results suggest that the relationship between the built environment and well-being is likely more complex than accounted for in this study. The complexity of the relationship might be explained by an ecological model, which describes older adults’ vulnerability to environmental demands. From this perspective, person-environment fit moderates psychological well-being through residential satisfaction and perceived social support (Cvitkovich & Wister, 2013; Lawton, 1977; Phillips et al., 2005).

Defining well-being differently or using different measures of the environment may have yielded different results. For example, in our model sense of community was part of psychosocial well-being. In other studies, it has have been treated as an independent variable and as a mediator in the relationship between the built environment and well-being as it can influence psychological well-being and impact the satisfaction of needs (Dong et al., 2014; Guo et al., 2021; Tang et al., 2017, 2018; Zhang et al., 2018). This calls for further investigation in future research, as our study could not explore these aspects independently.

### Impact of Subscales of Environment on Well-Being

The results of Model 3 highlight the critical role of objectively measured acceptability, sensory support, and dementia-friendly design in shaping well-being. The negative impact of low acceptability and support of people with dementia highlights the need for environments that accommodate cognitive and functional impairments, which can be achieved by natural environments and multisensory rooms as they are examples of cognitively stimulating spaces (Finlay et al., 2021; Freeman et al., 2019; Magnussen et al., 2021; van Hoof et al., 2010). Meanwhile, the positive effect of sensory support suggests that enhancing multisensory experiences can improve overall well-being (Minucciani & Saglar Onay, 2020). These findings underscore the importance of designing spaces that are not only functional but also supportive of diverse sensory and cognitive needs, which are higher-level needs based on Maslow's hierarchy of needs (Maslow, 1943). Respondents prioritize accessibility and aesthetic attributes in their perception of the environment. Other studies have shown that aesthetic elements like natural light, pleasant scenes and sounds, and green or blue spaces have a positive impact on older adults (Alidoust & Bosman, 2017; Falk et al., 2009; Finlay et al., 2021; Lipovac et al., 2022; Magnussen et al., 2021; Schmidt et al., 2021). These factors outweigh physical elements like windows, light, or air quality, suggesting that psychological comfort and a sense of home are key in designing supportive environments. However, the impact of aesthetic aspects on well-being has not been studied extensively enough to confirm their influence with great certainty.

*These findings underscore the importance of designing spaces that are not only functional but also supportive of diverse sensory and cognitive needs, which are higher-level needs based on Maslow's hierarchy of needs (Maslow, 1943). Respondents prioritize accessibility and aesthetic attributes in their perception of the environment.*

#### Limitations

Our results are limited due to sample size, which constrained our ability to include comprehensive demographic data. Excluding residents with dementia, visual impairment, mobility limitations, or dependency on others limited our results’ generalizability, as many retirement home residents with these conditions live among eligible participants. Choosing coastal retirement homes may also limit generalizability, as the environment, including views of blue spaces, which may impact well-being (Finlay et al., 2015).

Additionally, the questions added to LSNS-6 and the ARHQ tool have not been psychometrically validated, which may affect the reliability of our findings, and it is likely that revisions to the scale could simplify the procedure for participants and yield better results. Moreover, since sense of community, social network, psychological well-being, and needs were components of the latent variable for psychosocial well-being, they were not independently tested as mediators.

### Recommendations for Future Studies

Even though we controlled for various covariates, future studies should consider incorporating more culture-based variables, as well-being may be influenced by cultural factors (Ingersoll-Dayton, 2011). Since marital status and living arrangements affect well-being, further research should explore the role of social capital, social networks, and loneliness in retirement communities in moderating psychosocial well-being. Place attachment and identity are important factors when connecting well-being to the built environment and should be included in future studies as well. Additionally, qualitative approaches could offer nuanced insights into how residents perceive their environment and how these perceptions influence overall well-being. Furthermore, the connection between the environment and sense of community, and the environment and needs, as independent variables, should be investigated, as sense of community is a key part of the social aspect of the meaning of home, and satisfied needs contribute to well-being (Altomonte et al., 2020; Bigonnesse et al., 2014; Buckley, 2022).

## Conclusion

This study investigated the role of objectively and subjectively assessed retirement home interiors on retirement home residents’ well-being along the Slovenian coast. The results revealed a complex relationship between psychosocial well-being and the built environment in these retirement homes. The findings suggest that well-being is a significant contributor to how individuals perceive their built environment. Similarly, objectively measured acceptability, sensory support, and dementia-friendly design, along with subjectively evaluated homelike attributes, significantly affect well-being. Future research may explore the longitudinal effects of environmental changes on well-being, focusing on how multisensory environments and psychologically comforting spaces can enhance the well-being of retirement homes’ occupants.

*The findings suggest that well-being is a significant contributor to how individuals perceive their built environment. Similarly, objectively measured acceptability, sensory support, and dementia-friendly design, along with subjectively evaluated homelike attributes, significantly affect well-being.*

## Supplemental Material

sj-docx-1-her-10.1177_19375867251343909 - Supplemental material for Linking Older Adults’ Psychosocial Well-Being With Objective and Perceived Environments in Slovenia Supplemental material, sj-docx-1-her-10.1177_19375867251343909 for Linking Older Adults’ Psychosocial Well-Being With Objective and Perceived Environments in Slovenia by Mateja Erce Paoli and Michael D. Burnard in HERD: Health Environments Research & Design Journal

## Implications for Practice

- Actively involve older adults as participants in the design or refurbishment process by asking about their specific needs and priorities.
- Incorporating older adults' input helps ensure the environment aligns with what matters most to them, positively shaping residents' perceptions of their environment.
- Building standards should be set high and grounded in evidence-based research, as these standards directly influence residents' perceptions of their surroundings.
- Acceptability, sensory and dementia support features, accessibility, and aesthetic qualities appear to be the most important environmental factors influencing older adults' wellbeing.
- Relationships between environment and wellbeing are complex and remains ambiguous.
