# Supplementary material for: Linking Older Adults’ Psychosocial Well-Being With Objective and Perceived Environments in Slovenia
Source: HERD. 2025 Jun 25;18(4):69–84. doi: 10.1177/19375867251343909 (PMC12460924; doi:10.1177/19375867251343909)
Supplement: sj-docx-1-her-10.1177_19375867251343909 - Supplemental material for Linking Older Adults’ Psychosocial Well-Being With Objective and Perceived Environments in Slovenia [file sj-docx-1-her-10.1177_19375867251343909.docx]

Table S1. ARHQ questionnaire.

**Room section**

The following questions are all related only to your room and NOT to the retirement home as a whole. Please keep in mind we are asking you only about the room and not about the other nearby spaces, such as the bathroom.

|  | Items | Completely disagree | Disagree | Neither Agree nor Disagree | Agree | Completely agree |
| --- | --- | --- | --- | --- | --- | --- |
| 1 | The floor in my room looks nice. |  |  |  |  |  |
| 2 | The ceiling in my room looks nice. |  |  |  |  |  |
| 3 | The walls in my room look nice. |  |  |  |  |  |
| 4 | The furniture in my room looks nice. |  |  |  |  |  |
| 5 | My room has high-quality furniture. |  |  |  |  |  |
| 6 | The furniture in my room is practical. |  |  |  |  |  |
| 7 | I like the materials the furniture in my room is made of. |  |  |  |  |  |
| 8 | I like the materials of the window coverings (e.g., curtains, blinds, shutters) in my room are made of. |  |  |  |  |  |
| 9 | My room offers a variety of interesting things to see, hear, smell, or touch. |  |  |  |  |  |
| 10 | The view from my room window is nice. |  |  |  |  |  |
| 11 | Through my window, I can see activities of people or animals. |  |  |  |  |  |
| 12 | In my room, I can often hear sounds of nature, such as birds, water, or wind. |  |  |  |  |  |
| 13 | When I am in my room, I am usually not bothered by sounds coming from outside of my room. |  |  |  |  |  |
| 14 | In my room, I am generally not bothered by smells. |  |  |  |  |  |
| 15 | Most of the time the amount of light in the room allows me to do what I want. |  |  |  |  |  |
| 16 | Most of the time the light in my room is pleasant. |  |  |  |  |  |
| 17 | I have the possibility to customize my room according to my preferences. |  |  |  |  |  |
| 18 | In my room, there is enough space to move around comfortably. |  |  |  |  |  |
| 19 | My room enables enough privacy. |  |  |  |  |  |
| 20 | My room evokes a homelike feeling. |  |  |  |  |  |
| 21 | My room is decorated nicely (e.g., with plants or images). |  |  |  |  |  |
| 22 | My room is relaxing most of the time. |  |  |  |  |  |
| 23 | In the warmer period of the year, the humidity level in my room is suitable most of the time. |  |  |  |  |  |
| 24 | In the warmer period of the year, the temperature in my room is suitable most of the time. |  |  |  |  |  |
| 25 | In the warmer period of the year, the air in my room is fresh most of the time. |  |  |  |  |  |
| 26 | In the cooler period of the year, the humidity level in my room is suitable most of the time. |  |  |  |  |  |
| 27 | In the cooler period of the year, the temperature in my room is suitable most of the time. |  |  |  |  |  |
| 28 | In the cooler period of the year, the air in my room is fresh most of the time. |  |  |  |  |  |
| 29 | The distance I need to travel from my bed to the bathroom is short enough. |  |  |  |  |  |
| 30 | My room is tidy. |  |  |  |  |  |
| 31 | My room is clean. |  |  |  |  |  |
| 32 | My room looks nice. |  |  |  |  |  |

**Retirement home section**

The upcoming questions focus on the retirement home, considering it from the perspective of your room.

|  |  | Completely disagree | Disagree | Neither Agree nor Disagree | Agree | Completely agree |
| --- | --- | --- | --- | --- | --- | --- |
| 1 | From my room, it is easy to navigate to different spaces in the retirement home. |  |  |  |  |  |
| 2 | The routes I take most often from my room to other parts of the retirement home are safe. |  |  |  |  |  |
| 3 | From my room, it does not take a lot of effort to reach different spaces in the retirement home. |  |  |  |  |  |
| 4 | When going from my room to different spaces of the retirement home, there is enough equipment on the way that helps with moving around, such as handrails, fences, and seating. |  |  |  |  |  |
| 5 | On my way from my room to the different areas of the retirement home, I only pass through areas that are pleasant. |  |  |  |  |  |

Table S2. Included domains from EVOLVE tool.

| Domains | Nu. of items | Example |
| --- | --- | --- |
| Universal needs of all age groups | | |
| Personal realization and choice | 34 | The bedroom is separate from other rooms. |
| Dignity and privacy | 8 | Is a WC which can be accessed without going through a bedroom. |
| Comfort and control | 46 | The shading devices can be operated by people using the bathroom. |
| Personal care | 16 | The shower is a walk-in shower. |
| Social support outside building | 4 | The bedroom window(s) overlooks outdoor spaces where there is human activity. |
| Needs of older adults | | |
| Accessibility | 29 | There is more than 1500mm space on one side of the bed. |
| Physical support | 34 | The bedroom electrical sockets can be reached without having to move bedroom furniture. |
| Sensory support | 71 | The bedroom is decorated in a light colour with matt finish. |
| Dementia support | 42 | The bathroom door colour contrasts with the surrounding wall colour. |
| Health and safety | 35 | The bedroom light switches are visible in the dark. |
| Security | 4 | The bathroom window(s) has locks. |
| Working care | 6 | There is more than 600mm round the sides of the washbasin to allow a carer to be present. |

*Note.* Four answers were possible: yes, no, not in use, not applicable.

Table S3. Covariates with levels.

| Covariates | Levels |
| --- | --- |
| Age | 1. 60 to 64  2. 65 to 69  3. 70 to 74  4. 75 to 79  5. 80 to 84  6. 85 to 89  7. 90 or more |
| Reasons for choosing a retirement home | 0. Health condition  1. Unsettled family situations (e.g. social distress, violence, family disputes)  2. Loneliness  3. Desire for an orderly life  4. Other: |
| Time to adapt | 1. Less than a month  2. One month to two months  3. Three to four months  4. Five to six months  5. More than six months  6. I haven't adjusted yet |
| Chronic diseases | Number |
| Education level | 0. Preschool education  1. Elementary school  2. Lower secondary education (e.g. secondary vocational or professional school)  3. Higher secondary education (e.g. high school)  4. Higher education (6/1)  5. Higher education (6/2)  6. Diploma or equivalent level (6/2)  7. Masters or equivalent level (7 or 8/1)  8. Doctoral or equivalent level (8/2) |
| Marital status | 1. Married  2. Married but living apart  3. In an extramarital relationship  4. Divorced  5. Widower/widow  6. Single |
| Number of roommates | Number |
| Type of building lived in most of their life | 1. Single-family houses  2. Multi-apartment houses  3. Apartment |
| Residency location | 1. City  2. Suburban  3. Village |
| Income | 1. I have a much lower income than other people my age  2. I have a lower income than other people my age  3. I have a similar income to other people my age  4. I have a slightly higher income than other people my age  5. I have a much higher income than other people my age |
| Having a balcony | 1. Yes  2. No |
| Sky orientation | 1. North  2. East  3. South  4. West |
| Number of plants | Number |

Table S4. Definition of terms from Figure 2.

| Terms from Figure 2 | Explanation |
| --- | --- |
| Obj. env. | Objective measurement of environment (EVOLVE) |
| Married, living apart | Married, but living apart |
| Reason – unsettled fam. situations | Unsettled family situations |
| Reason – loneliness | Loneliness as a reason for moving to a retirement home |
| Reason – orderly life | Desire for an orderly life as a reason |
| Reason – other | Different reason for moving to a home besides health condition, unsettled family situations, loneliness, or desire for an orderly life |
| Ret. Home B | Retirement home B |
| Ret. Home C | Retirement home C |
| Multi-fam. house | Multi-family house |
| Nu. of diseases | Number of diseases |
| Wellbeing | Psychosocial wellbeing |
| Psy. wellbeing | Psychological wellbeing |
| Soc. support | Social support |

Table S5. Factorial structure of the ARHQ.

| Variable/ factors | Aesthetic qualities of a room | Visual qualities of a room | Indoor air quality | Control over environment |
| --- | --- | --- | --- | --- |
| The floor in my room looks nice. | **0.65** | -0.05 | 0.08 | -0.21 |
| The ceiling in my room looks nice. | **0.62** | 0.09 | 0.06 | 0.00 |
| The walls in my room look nice. | **0.45** | 0.12 | 0.22 | -0.02 |
| The furniture in my room looks nice. | **0.66** | 0.01 | 0.03 | 0.21 |
| My room has high-quality furniture. | **0.47** | 0.24 | -0.06 | 0.01 |
| The furniture in my room is practical. | 0.24 | -0.01 | -0.04 | 0.35 |
| I like the materials the furniture in my room is made of. | 0.37 | 0.11 | -0.02 | -0.08 |
| I like the materials of the window coverings (e.g., curtains, blinds, shutters) in my room are made of. | 0.34 | 0.27 | -0.14 | -0.05 |
| My room offers a variety of interesting things to see, hear, smell, or touch. | 0.32 | 0.10 | -0.14 | 0.15 |
| The view from my room window is nice. | -0.04 | **0.76** | -0.02 | 0.04 |
| Through my window, I can see activities of people or animals. | 0.03 | **0.59** | 0.13 | -0.01 |
| In my room, I can often hear sounds of nature, such as birds, water, or wind. | 0.09 | 0.38 | -0.07 | 0.05 |
| When I am in my room, I am usually not bothered by sounds coming from outside of my room. | 0.29 | 0.33 | 0.24 | -0.01 |
| In my room, I am generally not bothered by smells. | 0.09 | 0.13 | -0.18 | 0.31 |
| Most of the time the amount of light in the room allows me to do what I want. | 0.03 | **0.70** | -0.01 | 0.04 |
| Most of the time the light in my room is pleasant. | 0.09 | **0.48** | 0.00 | 0.12 |
| I have the possibility to customize my room according to my preferences. | -0.07 | 0.10 | -0.09 | **0.41** |
| In my room, there is enough space to move around comfortably. | 0.08 | 0.12 | 0.09 | **0.58** |
| My room enables enough privacy. | -0.13 | 0.08 | 0.04 | **0.78** |
| My room evokes a homelike feeling. | 0.35 | -0.14 | 0.03 | 0.39 |
| My room is decorated nicely (e.g., with plants or images). | 0.24 | 0.05 | 0.04 | 0.39 |
| My room is relaxing most of the time. | **0.51** | -0.13 | 0.00 | 0.39 |
| In the warmer period of the year, the humidity level in my room is suitable most of the time. | 0.04 | 0.16 | **0.62** | -0.06 |
| In the warmer period of the year, the temperature in my room is suitable most of the time. | 0.07 | 0.29 | **0.40** | 0.01 |
| In the warmer period of the year, the air in my room is fresh most of the time. | -0.03 | -0.03 | **0.88** | -0.01 |
| In the cooler period of the year, the humidity level in my room is suitable most of the time. | 0.05 | -0.04 | **0.43** | 0.10 |
| In the cooler period of the year, the temperature in my room is suitable most of the time. | 0.11 | 0.07 | 0.11 | 0.25 |
| In the cooler period of the year, the air in my room is fresh most of the time. | 0.05 | -0.08 | **0.54** | 0.15 |
| The distance I need to travel from my bed to the bathroom is short enough. | 0.09 | 0.18 | 0.11 | 0.05 |
| My room is tidy. | **0.60** | 0.09 | -0.01 | -0.01 |
| My room is clean. | **0.69** | 0.06 | -0.04 | -0.16 |
| My room looks nice. | **0.52** | -0.08 | 0.22 | 0.18 |

*Note.* CFI = 0.888, TLI = 0.866, RMSEA = 0.068, SRMR = 0.069, χ^2^ (158) = 254.573, p = 0.000

| Variable/ factors | Accessibility |
| --- | --- |
| From my room, it is easy to navigate to different spaces in the retirement home. | **0.70** |
| The routes I take most often from my room to other parts of the retirement home are safe. | **0.75** |
| From my room, it does not take a lot of effort to reach different spaces in the retirement home. | **0.58** |
| When going from my room to different spaces of the retirement home, there is enough equipment on the way that helps with moving around, such as handrails, fences, and seating. | **0.41** |
| On my way from my room to the different areas of the retirement home, I only pass through areas that are pleasant. | 0.37 |

*Note.* CFI = 1.00, TLI = 1.055, RMSEA = 0.000 SRMR = 0.019, χ^2^ (4) = 1.406, p = 0.843
